# White Matter Structural Connectivity Is Not Correlated to Cortical Resting-State Functional Connectivity over the Healthy Adult Lifespan

**DOI:** 10.3389/fnagi.2017.00144

**Published:** 2017-05-18

**Authors:** Adrian Tsang, Catherine A. Lebel, Signe L. Bray, Bradley G. Goodyear, Moiz Hafeez, Roberto C. Sotero, Cheryl R. McCreary, Richard Frayne

**Affiliations:** ^1^Department of Radiology, University of CalgaryCalgary, AB, Canada; ^2^Hotchkiss Brain Institute, University of CalgaryCalgary, AB, Canada; ^3^Seaman Family MR Research Centre, Foothills Medical Centre, Alberta Health ServicesCalgary, AB, Canada; ^4^Alberta Children's Hospital Research Institute, University of CalgaryCalgary, AB, Canada; ^5^Child and Adolescent Imaging Research Program, Alberta Children's Hospital, Alberta Health ServicesCalgary, AB, Canada

**Keywords:** multi-modal analysis, structural connectivity, functional connectivity, lifespan, aging

## Abstract

Structural connectivity (SC) of white matter (WM) and functional connectivity (FC) of cortical regions undergo changes in normal aging. As WM tracts form the underlying anatomical architecture that connects regions within resting state networks (RSNs), it is intuitive to expect that SC and FC changes with age are correlated. Studies that investigated the relationship between SC and FC in normal aging are rare, and have mainly compared between groups of elderly and younger subjects. The objectives of this work were to investigate linear SC and FC changes across the healthy adult lifespan, and to define relationships between SC and FC measures within seven whole-brain large scale RSNs. Diffusion tensor imaging (DTI) and resting-state functional MRI (rs-fMRI) data were acquired from 177 healthy participants (male/female = 69/108; aged 18–87 years). Forty cortical regions across both hemispheres belonging to seven template-defined RSNs were considered. Mean diffusivity (MD), fractional anisotropy (FA), mean tract length, and number of streamlines derived from DTI data were used as SC measures, delineated using deterministic tractography, within each RSN. Pearson correlation coefficients of rs-fMRI-obtained BOLD signal time courses between cortical regions were used as FC measure. SC demonstrated significant age-related changes in all RSNs (decreased FA, mean tract length, number of streamlines; and increased MD), and significant FC decrease was observed in five out of seven networks. Among the networks that showed both significant age related changes in SC and FC, however, SC was not in general significantly correlated with FC, whether controlling for age or not. The lack of observed relationship between SC and FC suggests that measures derived from DTI data that are commonly used to infer the integrity of WM microstructure are not related to the corresponding changes in FC within RSNs. The possible temporal lag between SC and FC will need to be addressed in future longitudinal studies to better elucidate the links between SC and FC changes in normal aging.

## Introduction

It is widely accepted that the normal human aging process involves changes in the brain's structural and functional connections. Understanding these changes will greatly improve our ability to diagnose and treat age-related neurodegenerative diseases, such as Alzheimer's Disease (AD), amyotrophic lateral sclerosis (ALS), and Parkinson's Disease (PD) (Pievani et al., 2014; Iturria-Medina and Evans, 2015; Gao and Wu, 2016). Non-invasive neuroimaging techniques including diffusion tensor imaging (DTI) and resting-state functional MRI (rs-fMRI) permit the investigation of white and gray matter connectivity in the brain. Metrics derived from DTI are used to quantify the white matter (WM) microstructure [termed structural connectivity (SC)], and correlations of the blood oxygen level dependent (BOLD) time signals computed from rs-fMRI are used to quantify the strength of resting state functional connections between distinct gray matter (GM) regions [termed functional connectivity (FC)].

Several studies have independently used DTI and rs-fMRI to demonstrate changes in SC and FC over the healthy human lifespan. In general, DTI studies have observed a non-linear inverted U-shaped trajectory association between age and fractional anisotropy (FA), and an U-shaped trajectory (opposite to FA) for axial, mean, and radial diffusivity (AD, MD, RD, respectively; Westlye et al., 2010; Lebel et al., 2012; Chen et al., 2013). Furthermore, previous studies have also used DTI to demonstrate that the degree of age-related cognitive decline correlates with WM microstructural alterations (Madden et al., 2012; Hawkins et al., 2015). On the other hand, rs-fMRI studies have reported both negative and positive (as well as both linear and non-linear) associations between age and FC, which were dependent on the brain region under investigation (Wang et al., 2012; Cao et al., 2014; Fjell et al., 2015a). In addition, rs-fMRI studies have also demonstrated that cognitive decline is related to decreased FC in the salience network (Onoda et al., 2012). As functionally linked cortical regions are connected anatomically via the underlying WM architecture (van den Heuvel et al., 2009), investigating SC and FC simultaneously to determine their interrelationship has the potential to provide a better, more comprehensive, understanding of the brain changes associated with aging.

The relationship between SC and FC, however, is not straightforward. For example, in one case following complete commissurotomy, FC was preserved across hemispheres between regions associated with the default mode network (Uddin et al., 2008). Similarly, another study showed no statistical differences in inter-hemispheric FC between subjects with complete agenesis of the corpus callosum and normal controls of comparable age, gender and IQ (Tyszka et al., 2011). Hence, multiple underlying (and possibly indirect) structural architectures must exist to support functional networks. Indeed, studies of healthy subjects have demonstrated strong FC between cortical regions with direct structural (i.e., WM) connections, as well as between regions in the absence of a direct WM pathway (Koch et al., 2002; Honey et al., 2009). Furthermore, Honey et al. (2009) also demonstrated that FC between indirectly connected regions was mediated by WM tract distance. Hence, these studies provide evidence that functionally connected cortical regions of a resting-state network (RSN) are either connected anatomically via a direct WM pathway or indirectly via WM tracts through one or more intermediate cortical or subcortical regions. However, whether the change in SC and FC measures with age are correlated within multiple large-scale RSNs across the healthy lifespan remains unknown or poorly understood.

The first study to adopt a multi-modal analysis of SC and FC demonstrated that FC between medial prefrontal cortex and the posterior cingulate/retrosplenial cortex (regions associated with the default mode network) was positively correlated with mean FA of the superior longitudinal fasciculus and cingulum WM tracts in elderly subjects (Andrews-Hanna et al., 2007). Subsequent multi-modal studies that compared data between two groups of subjects (i.e., young vs. elderly) demonstrated that FA and MD were significantly correlated with FC (Fjell et al., 2015b; Marstaller et al., 2015). To our knowledge, only three recent studies have examined SC and FC data from healthy subjects across the adult lifespan (Betzel et al., 2014; Lee et al., 2015; Fjell et al., 2016). Using a graph theory analysis approach, Betzel et al. showed that on average, FC remained relatively constant over the adult lifespan for regions with direct structural connections, but the change in FC with age was progressively greater as the structural connection distance between regions increased. Lee et al. demonstrated significant increases in SC and FC with age between prefrontal cortex and posterior regions of the parietal and temporal lobes, suggesting the brain adapts to neural challenges during normal aging. The study by Fjell et al. demonstrated a weak relationship between SC and FC measures for certain major WM tracts and their associated regions of the default mode network. While these studies provided important insights into SC and FC changes associated with normal aging, it remains unclear if WM microstructural changes over the adult lifespan are correlated with the corresponding FC changes within multiple large-scale RSNs. As there are only few studies in literature that investigated the relationship between SC and FC over the adult lifespan, and the results reported are related to certain specific GM regions and WM tracts, a study that investigates more broadly across multiple commonly described RSNs and the associated WM tracts is warranted in normal subjects over a wide age span.

In this study, we hypothesized that WM SC (using measurements of MD, FA, mean tract length, and number of streamlines) derived from DTI data across the adult lifespan are correlated with corresponding FC measures within seven commonly described large-scale RSNs. The aims of this work were to investigate (1) the relationship in SC and FC measures with age, (2) sex differences of these measures with age, and (3) the relationship between SC and FC measures, within seven commonly described RSNs in healthy participants across the adult lifespan.

## Materials and methods

### Participants

As part of an on-going normative study (the Calgary Normative Study) that was approved by the University of Calgary Research Ethics Board, healthy community-dwelling participants were recruited, initially screened over the phone, and only those who indicated no known neurological diseases and no contraindications to MR imaging were enrolled in the study. Informed written consent and basic medical history were obtained from each eligible participant prior to imaging. Participants were excluded from the analysis if there were medically significant incidental findings found on their MR images. In addition to MR imaging, the Montreal Cognitive Assessment (MoCA) was administered to each participant as a brief screening tool for mild cognitive impairment or dementia. Two hundred and twenty-one participants provided data for this study. Five subjects were excluded from our analysis due to incidentally discovered, potentially medically significant findings (1 subject), incomplete scan or missing data (2 subjects), or poor quality DTI data (2 subjects). Furthermore, 39 (male/female = 17/22) participants who obtained scores of <26 (out of 30) on the MoCA were excluded, as this falls outside the normal range. Subsequently, 177 subjects (aged 18–87 years; male/female = 69/108; Table 1) were included in the study.

**Table 1:** **Summary of study subject characteristics**.

**Age**	**18–29**	**30–39**	**40–49**	**50–59**	**60–69**	**70–87**
Number	38	30	27	33	30	19
Male/Female	15/23	14/16	8/19	16/17	11/19	5/14
MoCA score (mean ± *SD*)	28.9 ± 1.2	28.4 ± 1.2	28.2 ± 1.2	28.1 ± 1.5	27.9 ± 1.2	27.3 ± 1.3

### Image acquisition

MR imaging was performed on a 3.0 T clinical scanner (Discovery MR750; GE Healthcare, Waukesha, WI) using a 12-channel phased-array head coil. The image acquisition protocol included DTI, rs-fMRI, T2-weighted FLAIR, and T1-weighted imaging sequences. DTI acquisition employed a single-shot spin-echo echo-planar imaging (EPI) sequence [echo time (TE) = 80 ms; repetition time (TR) = 9,000 or 10,000 ms; 48–52 contiguous 3-mm thick slices; field of view (FOV) = 240 × 240 mm; acquired matrix = 80 × 80 interpolated to 256 × 256; reconstructed in-plane resolution = 0.94 × 0.94 mm] with diffusion sensitizing gradients applied in 31 non-collinear directions (*b* = 1000 s/mm^2^) and 4 *b* = 0 s/mm^2^ volumes. rs-fMRI acquisition consisted of a single-shot gradient-echo EPI sequence (TE = 30 ms; TR = 2,000 ms; 37 contiguous 3.8-mm thick slices; acquired matrix = 64 × 64; FOV = 240 × 240 mm; reconstructed voxel size = 3.8 mm isotropic) and acquired 150 whole brain volumes over a 5-min interval. T2-weighted FLAIR images were acquired using an inversion recovery prepared fast spin echo sequence [flip angle = 111°; inversion time (TI) = 2,250 ms; TE = 141.4 ms; TR = 9,000 ms; 48 contiguous 3-mm thick slices, FOV = 240 × 240 mm; reconstructed voxel size = 0.94 × 0.94 mm]. T1-weighted anatomical images were acquired using a 3D inversion recovery prepared spoiled gradient-echo sequence [flip angle = 8°; inversion time (TI) = 650 ms; TE = 2.5 ms; TR = 6.3 ms; acquired matrix size = 256 × 256 × 166; phase FOV = 85%; reconstructed voxel size = 1 mm isotropic].

### Image processing

#### Identification of cortical regions

**Figure 1:**
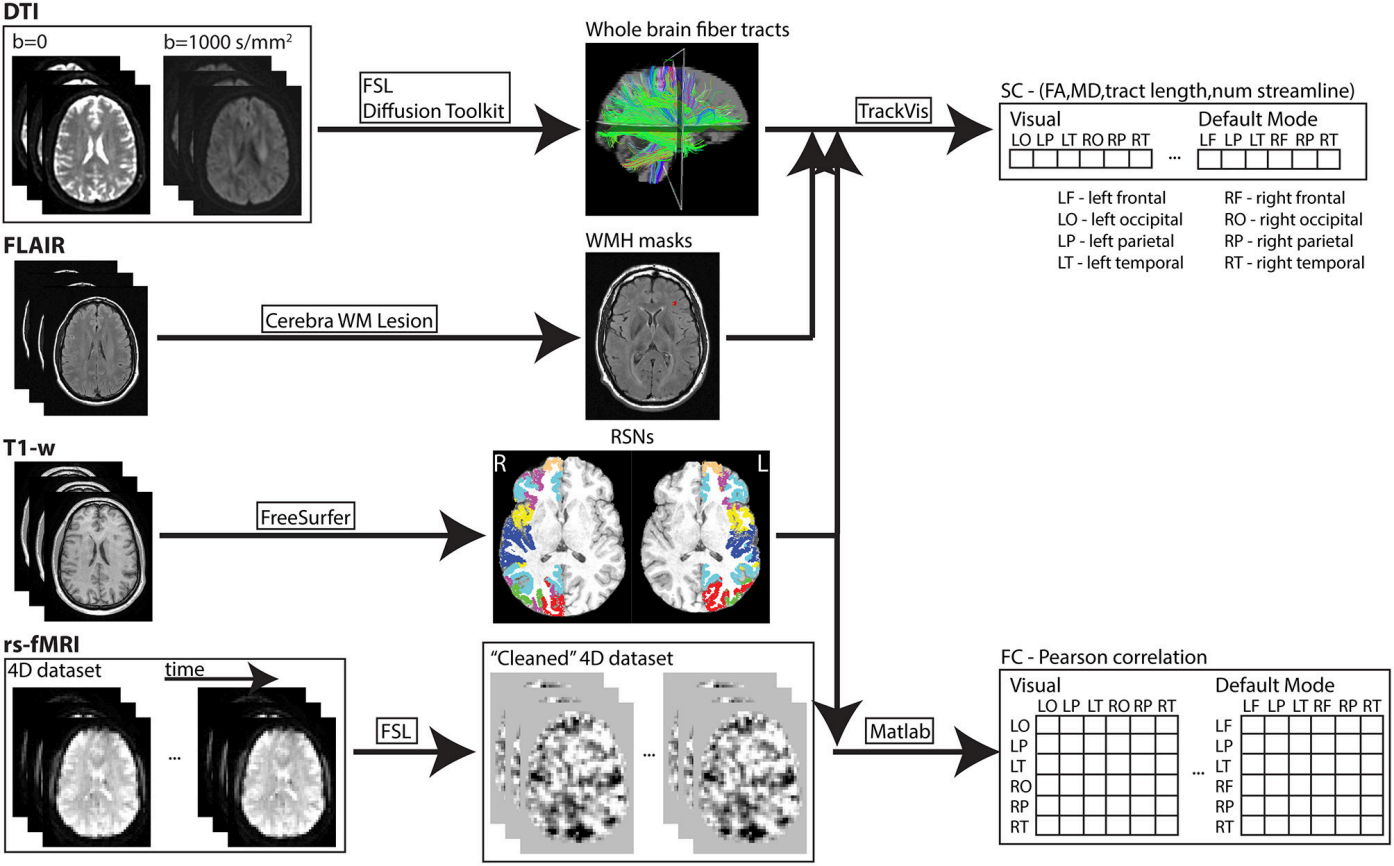
**In-house developed pipeline used to process MR imaging data of each subject**. Data processing and analysis through this pipeline was performed on an iMac (2.9GHz quad-core Intel Core i5; 32GB 1600MHz DDR3 memory) and high performance computing clusters (https://www.westgrid.ca; used to generate cortical surface labels—explained above). SC and FC measures were derived for each resting-state network (RSN). Subsequently, the values of each connectivity measure were averaged in each network.

Images from each subject were processed using an in-house automated pipeline developed from freely available software packages and a semi-automated tool (Cerebra-WML; Gobbi et al., 2012) for WM hyper-intensity mask identification (see Figure 1). A cortical parcellation atlas (Yeo et al., 2011) was used to define cortical regions related to seven resting-state networks (RSNs). The atlas was constructed by processing rs-fMRI data acquired from 1,000 healthy participants and used a clustering algorithm to parcellate the cortex into multiple RSNs. There are other whole-brain atlases available with cortical parcellation including (Auzias et al., 2016; Fan et al., 2016) that can also be used in such multi-modal analysis, but the template by Yeo et al. was chosen as it is widely adopted by many previous studies. The coarse-resolution seven RSNs parcellation was chosen from the selected template atlas over the fine-resolution 17 networks parcellation simply to reduce computing resources and processing times. Nevertheless, the coarse parcellation accurately reflects seven distinct and commonly identified RSNs. Regions within each network were extracted based on four pre-specified anatomical lobes (i.e., frontal, parietal, temporal, occipital) and resulted in the identification of 40 cortical regions across both hemispheres. Specifically, cortical regions in each hemisphere for the seven networks were

1. visual network: occipital, intraparietal, inferior temporal regions;
2. somato-motor network: posterior frontal, anterior parietal, superior, and anterior temporal regions;
3. dorsal attention network: superior frontal, superior parietal, posterior temporal regions;
4. ventral attention network: inferior medial frontal, inferior lateral and superior medial parietal, superior temporal regions;
5. limbic network: inferior prefrontal, inferior temporal regions;
6. frontal-parietal network: lateral frontal, superior posterior parietal, inferior temporal regions; and
7. default mode network: medial lateral frontal, inferior parietal, lateral temporal regions.

Cortical surface labels using T1-weighted images were obtained from each subject (FreeSurfer; http://surfer.nmr.mgh.harvard.edu) and were used to transform each region from the atlas space to the subject native space. Subsequently, the 40 cortical regions were transformed to the subject DTI and rs-fMRI spaces for analysis.

#### Measurement of structural connectivity (SC)

**Figure 2:**
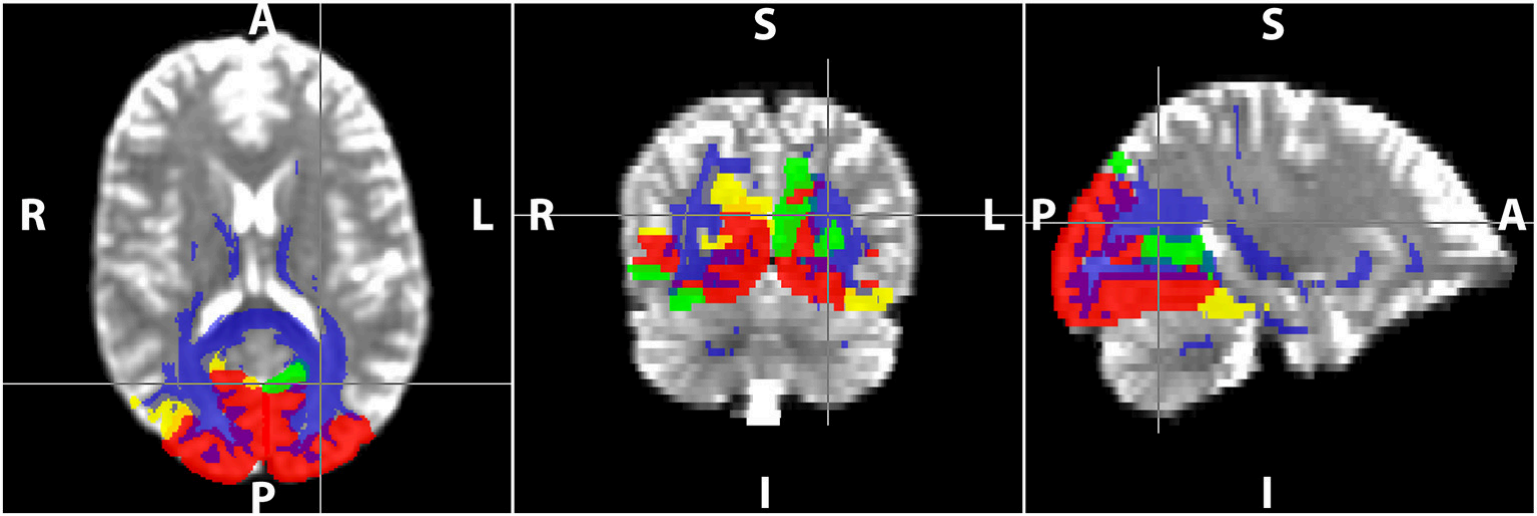
**Delineation of WM tracts using TrackVis in the visual resting-state network (RSN)**. The red, yellow, and green regions are the occipital, parietal, and temporal regions of the visual RSN, and the voxels in blue represent the WM voxels of all the tracts that are part of the RSN.

DTI data were first corrected for motion and eddy current distortion using FSL (FMRIB Software Library, version 5.0.8; http://www.fmrib.ox.ac.uk/fsl; Jenkinson et al., 2012). Maps of MD and FA were computed from the DTI data (Diffusion Toolkit; http://trackvis.org/dtk/), as well as statistics of mean tract length and number of streamlines were extracted from the tractography algorithm. Whole brain WM tracts were delineated by deterministic tractography using the second order Runge-Kutta algorithm (Basser et al., 2000) with the FA threshold set to 0.20 to exclude gray matter voxels and the angle threshold set to 35° to exclude tracks with sharp curvature. The 40 cortical regions from the template were first dilated and used as seeding/target regions to delineate WM tracts that either originate or terminate at each cortical GM region of the seven RSNs (TrackVis; http://trackvis.org). Example of the cortical seeding regions used for the visual network and the associated WM tracts delineated is shown in Figure 2. WM hyper-intensity voxels were excluded from FA and MD maps using the masks defined from FLAIR images.

#### Measurement of functional connectivity (FC)

The rs-fMRI data were first processed using the FSL package and included skull stripping (Brain Extraction Tool, BET; Smith, 2002), interleaved slice timing correction and motion correction (MCFLIRT algorithm; Jenkinson et al., 2002), spatial smoothing (6-mm full width at half maximum), and temporal high-pass filtering (>0.01 Hz) to eliminate low frequency artifacts. Noise components in the pre-processed data were removed using independent component analysis-based methods (FSL Xnoiseifier, FIX; Salimi-Khorshidi et al., 2014). In addition, the time-points of large motion perturbations in the original four-dimensional resting-state time series dataset were identified (FSL Motion Outliers) using a threshold of 0.2 mm (a stringent threshold for scrubbing; Power et al., 2014) applied for frame-wise displacement. A confound matrix was created for the large motion time-points and was included as additional event variable in the analysis to remove nuisance variables from the resting-state dataset (FMRI Expert Analysis Tool, FEAT). Cerebrospinal fluid (CSF) and WM masks were manually drawn on T1-weighted images from each subject and then transformed into the rs-fMRI image space. Six motion parameters and the average time series from CSF and WM masks were regressed out as nuisance variables (FEAT) from the pre-processed noise reduced (FIX) four-dimensional rs-fMRI dataset. The time-point volumes with motion greater than the threshold were removed from the “cleaned” rs-fMRI dataset. Average rs-fMRI-obtained BOLD signal of all voxels within individual regions from this processed dataset was computed for all time points for subsequent analysis (MATLAB R2015b; MathWorks, Natick, MA). Pearson correlation coefficients (r) of the averaged BOLD signal time series between pairs of regions in each network were converted to z-scores using Fisher's r-to-z transformation {z = 0.5 × ln [(1 + r)/(1 − r)]}.

### Statistical analyses

Average SC and FC measures were computed for each network, and these data were used for all the statistical analyses (SPSS version 22.0; IBM Corp, Armonk, NY). In all analyses, the critical value was chosen as α = 0.05 and multiple comparison corrections were applied across the seven networks using the Bonferroni method (Holm, 1979). Therefore, *p* < α/7 ≈ 0.007 were considered to be significant.

The following statistical tests (T1 to T4) were performed to address the three objectives of this study:

T1: Pearson correlations of each SC and FC measure with age in each network.
T2: Pearson correlations of each SC and FC measure with age in each network for male and female subjects separately. Following this, the slope of the linear regression line for each connectivity measure was tested for sex differences using *t*-tests provided in the *Real Statistics Resource Pack* software (Release 4.3; Zaiontz, 2013–2015).
T3: In addition to testing the age relationship with SC and FC measures above, we tested whether sex is a significant predictor of SC and FC using multiple linear regression. Both age and sex were added into a model (SC or FC = β_0_ + β_1_^*^ age + β_2_^*^ sex) using stepwise selection input method.
T4: Pearson correlations between each SC measure and FC in each network. In addition, partial correlations were performed to control for the effect of age that may affect both SC and FC.

## Results

### SC and FC changes with age (T1)

**Figure 3:**
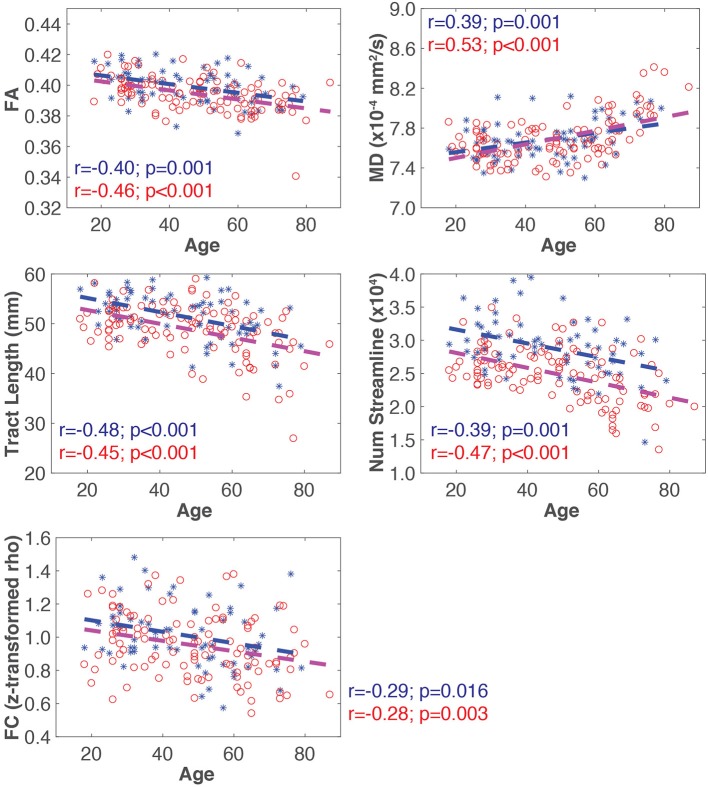
**Representative plots of SC and FC measures for all subjects (blue asterisk, male; red circle, female) for the ventral attention network**. The blue and red dash lines in each plot represent the linear trajectory to model the change of SC and FC measures with age for all male and female subjects, respectively. The Pearson correlation coefficient (*r*), and the corresponding uncorrected *p*-value, for each connectivity measure with age by sex is also shown.

A representative example of the relationships between SC and FC measure changes with age for the ventral attention network is shown in Figure 3. All four SC measures (i.e., FA, MD, mean tract length, number of streamlines) were significantly correlated with age in all networks (Table 2). FA, mean tract length, and number of streamlines were decreased with age, while MD was increased with age. On the other hand, there was a general trend of FC decrease with age in all networks. FC was significantly negatively correlated with age in five out of seven RSNs (somato-motor, dorsal attention, ventral attention, limbic, and frontal-parietal).

**Table 2:** **Pearson correlation coefficients (***r***) and slopes of the linear regression lines of SC (i.e., MD, FA, mean tract length, number of streamlines) and FC (i.e., Fisher's r-to-z transformed Pearson correlation coefficient) measures with age in each resting-state network (RSN)**.

**RSN**	**Connectivity measure**	**Slope**	***r***	***p***
Visual	MD (mm^2^/s)	6.53E-07	0.42	<0.001^*^
	FA	−4.08E-04	−0.54	<0.001^*^
	Tract length (mm)	−1.67E-01	−0.42	<0.001^*^
	Num streamlines	−1.12E+02	−0.42	<0.001^*^
	FC	−6.31E-04	−0.06	0.403
Somato-motor	MD (mm^2^/s)	3.81E-07	0.35	<0.001^*^
	FA	−1.94E-04	−0.31	<0.001^*^
	Tract length (mm)	−1.24E-01	−0.41	<0.001^*^
	Num streamlines	−1.28E+02	−0.37	<0.001^*^
	FC	−2.75E-03	−0.22	0.003^*^
Dorsal attention	MD (mm^2^/s)	2.76E-07	0.24	0.001^*^
	FA	−3.49E-04	−0.49	<0.001^*^
	Tract length (mm)	−1.94E-01	−0.50	<0.001^*^
	Num streamlines	−1.08E+02	−0.42	<0.001^*^
	FC	−2.28E-03	−0.24	0.001^*^
Ventral attention	MD (mm^2^/s)	6.11E-07	0.48	<0.001^*^
	FA	−2.99E-04	−0.44	<0.001^*^
	Tract length (mm)	−1.43E-01	−0.47	<0.001^*^
	Num streamlines	−1.17E+02	−0.43	<0.001^*^
	FC	−3.35E-03	−0.29	<0.001^*^
Limbic	MD (mm^2^/s)	5.94E-07	0.40	<0.001^*^
	FA	−3.88E-04	−0.50	<0.001^*^
	Tract length (mm)	−1.45E-01	−0.43	<0.001^*^
	Num streamlines	−1.04E+02	−0.49	<0.001^*^
	FC	−2.21E-03	−0.21	0.004^*^
Frontal-parietal	MD (mm^2^/s)	6.04E-07	0.47	<0.001^*^
	FA	−4.55E-04	−0.57	<0.001^*^
	Tract length (mm)	−2.12E-01	−0.62	<0.001^*^
	Num streamlines	−1.95E+02	−0.51	<0.001^*^
	FC	−1.90E-03	−0.21	0.006^*^
Default mode	MD (mm^2^/s)	6.90E-07	0.50	<0.001^*^
	FA	−4.28E-04	−0.57	<0.001^*^
	Tract length (mm)	−1.87E-01	−0.57	<0.001^*^
	Num streamlines	−2.86E+02	−0.54	<0.001^*^
	FC	−1.89E-03	−0.18	0.016

* *statistically significant after multiple comparison correction using the Bonferroni method*.

### Sex differences in SC and FC changes with age (T2)

The slope of the linear regression lines for all SC and FC measures with age were not statistically different between male and female subjects in any network (Table 3).

**Table 3:** **Pearson correlation coefficients (***r***) and slopes of the linear regression lines of SC (i.e., MD, FA, mean tract length, number of streamlines) and FC (i.e., Fisher's r-to-z transformed Pearson correlation coefficient) measures with age for male and female subjects in each resting-state network (RSN)**.

**RSN**	**Connectivity measure**	**Male**			**Female**				
		**Slope**	***r***	***p***	**Slope**	***r***	***p***	***t***	***p***
Visual	MD (mm^2^/s)	5.57E−07	0.39	0.001^*^	7.11E−07	0.43	<0.001^*^	0.68	0.499
	FA	−3.64E−04	−0.48	<0.001^*^	−4.23E−04	−0.57	<0.001^*^	−0.59	0.553
	Tract length (mm)	−7.57E−02	−0.21	0.083	−2.09E−01	−0.52	<0.001^*^	−2.43	0.016
	Num streamlines	−8.54E+01	−0.30	0.012	−1.18E+02	−0.50	<0.001^*^	−0.90	0.372
	FC	−1.17E−04	−0.01	0.921	−8.27E−04	−0.08	0.402	−0.45	0.655
Somato-motor	MD (mm^2^/s)	2.82E−07	0.26	0.029	4.37E−07	0.40	<0.001^*^	0.96	0.337
	FA	−1.65E−04	−0.25	0.043	−1.94E−04	−0.35	<0.001^*^	−0.32	0.753
	Tract length (mm)	−8.42E−02	−0.31	0.011	−1.37E−01	−0.46	<0.001^*^	−1.26	0.209
	Num streamlines	−1.03E+02	−0.30	0.013	−1.28E+02	−0.43	<0.001^*^	−0.53	0.596
	FC	−2.94E−03	−0.24	0.047	−2.52E−03	−0.20	0.039	0.21	0.832
Dorsal attention	MD (mm^2^/s)	1.51E−07	0.13	0.290	3.46E−07	0.30	0.002^*^	1.10	0.273
	FA	−2.69E−04	−0.36	0.003^*^	−3.79E−04	−0.56	<0.001^*^	−1.12	0.263
	Tract length (mm)	−1.29E−01	−0.34	0.004^*^	−2.23E−01	−0.58	<0.001^*^	−1.77	0.079
	Num streamlines	−8.40E+01	−0.31	0.010	−1.12E+02	−0.50	<0.001^*^	−0.79	0.433
	FC	−2.57E−03	−0.25	0.037	−1.97E−03	−0.22	0.022	0.41	0.681
Ventral attention	MD (mm^2^/s)	4.81E−07	0.39	0.001^*^	6.83E−07	0.53	<0.001^*^	1.14	0.256
	FA	−2.84E−04	−0.40	0.001^*^	−2.94E−04	−0.46	<0.001^*^	−0.11	0.912
	Tract length (mm)	−1.40E−01	−0.48	<0.001^*^	−1.38E−01	−0.45	<0.001^*^	0.06	0.950
	Num streamlines	−1.05E+02	−0.39	0.001^*^	−1.12E+02	−0.47	<0.001^*^	−0.22	0.829
	FC	−3.44E−03	−0.29	0.016	−3.12E−03	−0.28	0.003^*^	0.18	0.855
Limbic	MD (mm^2^/s)	5.00E−07	0.34	0.004^*^	6.42E−07	0.43	<0.001^*^	0.67	0.507
	FA	−3.35E−04	−0.43	<0.001^*^	−4.03E−04	−0.54	<0.001^*^	−0.65	0.517
	Tract length (mm)	−1.10E−01	−0.33	0.006^*^	−1.55E−01	−0.48	<0.001^*^	−0.96	0.339
	Num streamlines	−1.02E+02	−0.44	<0.001^*^	−9.75E+01	−0.54	<0.001^*^	0.15	0.881
	FC	−3.35E−04	−0.03	0.812	−3.25E−03	−0.34	<0.001^*^	−1.83	0.069
Frontal-parietal	MD (mm^2^/s)	5.30E−07	0.42	<0.001^*^	6.51E−07	0.49	<0.001^*^	0.66	0.510
	FA	−4.16E−04	−0.51	<0.001^*^	−4.63E−04	−0.59	<0.001^*^	−0.45	0.656
	Tract length (mm)	−1.87E−01	−0.60	<0.001^*^	−2.20E−01	−0.63	<0.001^*^	−0.78	0.439
	Num streamlines	−1.82E+02	−0.48	<0.001^*^	−1.87E+02	−0.57	<0.001^*^	−0.11	0.910
	FC	−1.62E−03	−0.17	0.172	−1.91E−03	−0.22	0.023	−0.20	0.840
Default mode	MD (mm^2^/s)	5.72E−07	0.44	<0.001^*^	7.58E−07	0.53	<0.001^*^	0.96	0.337
	FA	−4.18E−04	−0.54	<0.001^*^	−4.21E−04	−0.59	<0.001^*^	−0.03	0.975
	Tract length (mm)	−1.43E−01	−0.47	<0.001^*^	−2.05E−01	−0.62	<0.001^*^	−1.49	0.139
	Num streamlines	−2.51E+02	−0.45	<0.001^*^	−2.85E+02	−0.62	<0.001^*^	−0.53	0.598
	FC	−2.27E−03	−0.21	0.078	−1.59E−03	−0.15	0.112	0.41	0.682

*The t-statistics (female–male) and corresponding p-values are shown to indicate sex differences between the slopes*.

* *statistically significant after multiple comparison correction using the Bonferroni method*.

### Other predictors of SC and FC (T3)

Multiple linear regression analysis found that both the age and sex terms were significant for FA, mean tract length, and number of streamlines in all networks except in the visual network (only the age term was significant for FA). For MD and FC, only the age term was significant in all cases except in the visual network (FC remained unchanged with age; Table 4).

**Table 4:** **Multiple linear regression of SC (i.e., MD, FA, mean tract length, number of streamlines) and FC (i.e., Fisher's r-to-z transformed Pearson correlation coefficient) measures in each resting-state network (RSN)**.

**RSN**	**Connectivity measure**	**Model**	**Coefficients**					
			**β_1_**	***p***	**β_2_**	***p***	***R*^2^**
Visual	MD	a	6.53E−07	<0.001	–	–	0.173
	FA	a	−4.08E−04	<0.001	–	–	0.291
	Tract length (mm)	b	−1.62E−01	<0.001	2.17E+00	0.018	0.206
	Num streamlines	b	−1.07E+02	<0.001	2.70E+03	<0.001	0.261
	FC	–	–	–	–	–	–
Somato-motor	MD	a	3.81E−07	<0.001	–	–	0.124
	FA	b	−1.84E−04	<0.001	4.94E−03	0.001	0.151
	Tract length (mm)	b	−1.19E−01	<0.001	2.57E+00	<0.001	0.230
	Num streamlines	b	−1.19E+02	<0.001	4.35E+03	<0.001	0.277
	FC	a	−2.75E−03	0.003	–	–	0.048
Dorsal attention	MD	a	2.76E−07	0.001	–	–	0.057
	FA	b	−3.41E−04	<0.001	4.10E−03	0.011	0.264
	Tract length (mm)	b	−1.90E−01	<0.001	2.21E+00	0.011	0.275
	Num streamlines	b	−1.02E+02	<0.001	2.94E+03	<0.001	0.283
	FC	a	−2.28E−03	0.001	–	–	0.057
Ventral attention	MD	a	6.11E−07	<0.001	–	–	0.231
	FA	b	−2.91E−04	<0.001	4.18E−03	0.008	0.227
	Tract length (mm)	b	−1.38E−01	<0.001	2.34E+00	0.001	0.266
	Num streamlines	b	−1.09E+02	<0.001	3.72E+03	<0.001	0.341
	FC	a	−3.35E−03	<0.001	–	–	0.085
Limbic	MD	a	5.94E−07	<0.001	–	–	0.163
	FA	b	−3.79E−04	<0.001	4.33E−03	0.012	0.279
	Tract length (mm)	b	−1.39E−01	<0.001	2.90E+00	<0.001	0.244
	Num streamlines	b	−9.90E+01	<0.001	2.25E+03	<0.001	0.332
	FC	a	−2.21E−03	0.004	–	–	0.046
Frontal-parietal	MD	a	6.04E−07	<0.001	–	–	0.217
	FA	b	−4.46E−04	<0.001	4.24E−03	0.013	0.344
	Tract length (mm)	b	−2.08E−01	<0.001	1.68E+00	0.016	0.402
	Num streamlines	b	−1.85E+02	<0.001	4.87E+03	<0.001	0.402
	FC	a	−1.90E−03	0.006	–	–	0.043
Default mode	MD	a	6.90E−07	<0.001	–	–	0.246
	FA	b	−4.20E−04	<0.001	3.92E−03	0.013	0.353
	Tract length (mm)	b	−1.83E−01	<0.001	2.07E+00	0.003	0.359
	Num streamlines	b	−2.73E+02	<0.001	6.17E+03	<0.001	0.400
	FC	a	−1.89E−03	0.016	–	–	0.033

a SC/FC ~ β_1_^*^age.

b SC/FC ~ β_1_^*^age + β_2_^*^sex.

### Relationships between SC and FC (T4)

**Figure 4:**
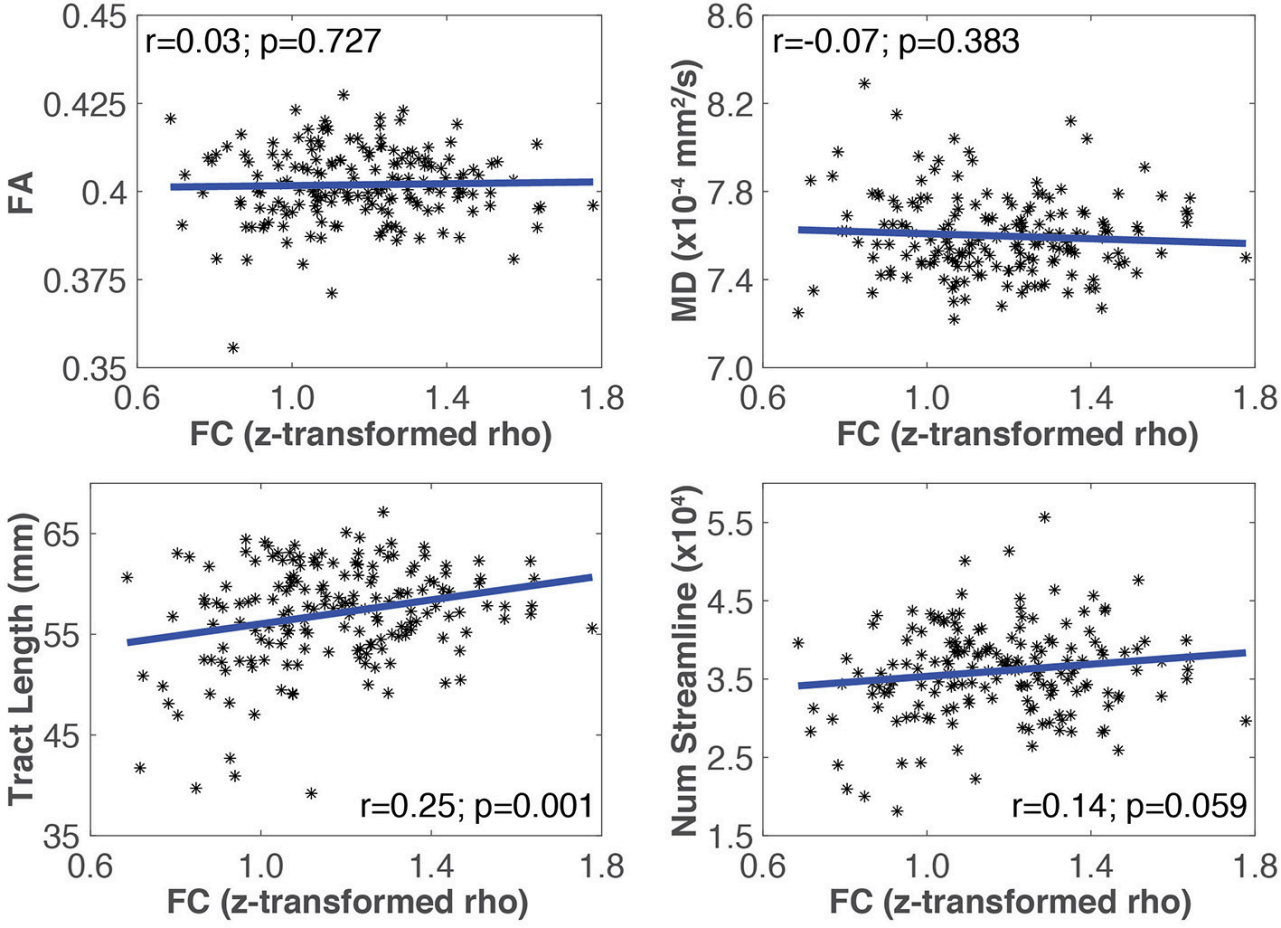
**Plots showing the linear relationship between SC (i.e., MD, FA, mean tract length, number of streamlines) and FC measures for the somato-motor network**. The Pearson correlation coefficient (*r*), and the corresponding uncorrected *p*-value, for each SC measure with FC is also shown. In this network, only mean tract length was significantly correlated with FC.

An example of Pearson correlations between SC and FC in the somato-motor network is shown in Figure 4. The four SC measures were not correlated with FC in all networks, except that the mean tract length in the somato-motor and ventral attention networks, and number of streamlines in the frontal-parietal network, were significantly related to FC (Table 5). Partial correlations did not reveal any significant relationship between SC and FC after controlling for the effect of age.

**Table 5:** **Pearson and partial (removing the age effect) correlation coefficients (***r***) of SC (i.e., MD, FA, tract length, num streamlines) with FC (i.e., Fisher's r-to-z transformed Pearson correlation coefficient) measures in each resting-state network (RSN)**.

**RSN**	**Structural connectivity measure**	**Pearson correlations**		**Partial correlations**	
		***r***	***p***	***r***	***p***
Visual	MD (mm^2^/s)	0.04	0.565	0.08	0.310
	FA	0.00	0.986	−0.04	0.578
	Tract length (mm)	0.04	0.563	0.02	0.806
	Num streamlines	0.09	0.238	0.07	0.362
Somato-motor	MD (mm^2^/s)	−0.07	0.383	0.01	0.875
	FA	0.03	0.727	−0.05	0.554
	Tract length (mm)	0.25	0.001^*^	0.18	0.018
	Num streamlines	0.14	0.059	0.07	0.377
Dorsal attention	MD (mm^2^/s)	0.03	0.706	0.09	0.229
	FA	0.11	0.160	−0.01	0.875
	Tract length (mm)	0.16	0.030	0.05	0.490
	Num streamlines	0.19	0.012	0.10	0.189
Ventral attention	MD (mm^2^/s)	−0.07	0.345	0.08	0.280
	FA	0.07	0.353	−0.07	0.368
	Tract length (mm)	0.21	0.004^*^	0.09	0.227
	Num streamlines	0.19	0.012	0.07	0.332
Limbic	MD (mm^2^/s)	−0.05	0.529	0.04	0.570
	FA	0.07	0.342	−0.04	0.579
	Tract length (mm)	0.01	0.918	−0.09	0.213
	Num streamlines	0.02	0.763	−0.10	0.209
Frontal-parietal	MD (mm^2^/s)	−0.01	0.917	0.10	0.175
	FA	0.11	0.166	−0.02	0.836
	Tract length (mm)	0.10	0.209	−0.04	0.569
	Num streamlines	0.21	0.005^*^	0.12	0.101
Default mode	MD (mm^2^/s)	−0.15	0.048	−0.07	0.359
	FA	0.14	0.071	0.04	0.595
	Tract length (mm)	0.12	0.111	0.02	0.778
	Num streamlines	0.14	0.068	0.05	0.513

* *Indicate statistical significance after multiple comparison corrections*.

## Discussion

In this study, the four SC measures demonstrated significant age-related changes in all seven RSNs across the healthy adult lifespan, while FC demonstrated significant age-related changes in four of the seven networks. In general, SC measures were not related to FC suggesting that WM microstructure as inferred from the SC measures derived from DTI data do not correlate with the corresponding cortical FC changes within a RSN.

### SC and FC changes with age

The observed correlations of SC and FC measures with age among all seven networks are consistent with published independent DTI (Westlye et al., 2010; Lebel et al., 2012; Chen et al., 2013) and rs-fMRI (Mevel et al., 2013; Cao et al., 2014; Fjell et al., 2015a) human brain aging studies. In these studies, other trajectories, such as quadratic or Poisson trajectories, were used to model age-related changes in SC (FA, MD, AD, RD) or FC across wider age ranges that included children and adolescents. Visual inspection of these published results for SC and FC changes in adulthood (≥18 years, the age range of participants in this study) shows the trend to more closely resemble a linear trajectory, which serves as a good approximation for SC and FC measures in this work. However, longitudinal and/or larger studies that include child and adolescent participants may better be able to elucidate the exact trajectories. Results for the other two SC measures in this study (i.e., mean tract length and number of streamlines) are also consistent with literature. A recent study that analyzed DTI data from 121 subjects between age 4 and 40 years demonstrated significant decrease in the number of streamlines, and the loss of streamlines occurred earlier in females than in males (Lim et al., 2015). The mean tract length decrease observed in this study was also consistent with two earlier studies that demonstrated WM fiber bundle length decrease in healthy adults over 50 years of age (Baker et al., 2014; Behrman-Lay et al., 2015).

### Sex differences in SC and FC

A significant effect of sex in the regression model was observed for FA, tract length, and number of streamlines across all networks except for FA in the visual network (Table 4). These three SC measures demonstrated significantly higher mean values in males. There was no significant difference between male and female subjects observed for MD. Our results agree with sex differences in FA (higher in male over female) of certain major WM tracts reported in previous studies (Hsu et al., 2008; Lebel et al., 2012). Higher mean FC was observed for males, though the sex term in the regression model was not significant for FC in any network. Sex differences in FC in healthy adults have been reported in previous studies using graph theory (Cao et al., 2014; Scheinost et al., 2015), however, not in previous rs-fMRI aging studies in normal adults that used the same FC metric as this work (i.e., z-transformed correlation of the averaged BOLD time signal; Wang et al., 2012; Fjell et al., 2015a).

### Relationship between SC and FC

In general, SC measures for WM tracts were not correlated with FC. However, some measures had correlations that were significant at a trend level, before multiple comparison correction (i.e., *p* < 0.05), suggesting that a weak relationship may exist between SC and FC in this sample (Table 5). Spatial averaging of metrics within a network could mask possible relationships between SC and FC in more spatially localized areas. Further analysis was performed to address this concern. All combinations of pairs of regions in each network were used as seed regions in the tractography algorithm, but only those with delineated WM tracts connecting them were considered in this analysis. The more spatially specific correlations of SC (i.e., FA, MD, tract length, number of streamlines) and FC for relevant pairs of regions in each network are shown in Table 6. Both network-averaged and more spatially specific results showed a lack of significant relationship between SC and FC, which is contrary to our hypothesis, and suggest that the changes in WM microstructure do not play a significant role in the corresponding changes in FC within large-scale RSNs.

**Table 6:** **Pearson correlation coefficients (***r***) of SC with FC measures for individual pairs of regions in each resting stating network (RSN)**.

		**MD vs. FC**		**FA vs. FC**		**Tract length vs. FC**		**Number of streamlines vs. FC**	
**RSN**	**Region pair**	***r***	***p***	***r***	***p***	***r***	***p***	***r***	***p***
Visual	lh_OL—lh_PL	0.12	0.114	−0.12	0.111	0.00	0.988	0.11	0.145
	lh_OL—lh_TL	0.00	0.957	0.11	0.144	0.05	0.492	0.14	0.067
	lh_OL—rh_OL	−0.15	0.045	0.06	0.455	0.11	0.139	0.19	0.013
	lh_OL—rh_PL	0.18	0.017	−0.02	0.797	0.07	0.337	−0.08	0.307
	lh_PL—rh_OL	−0.07	0.389	−0.09	0.240	−0.02	0.796	0.11	0.160
	lh_PL—rh_PL	−0.12	0.118	−0.02	0.826	0.07	0.379	0.02	0.806
	rh_OL—rh_PL	0.13	0.081	−0.18	0.018	−0.11	0.138	0.05	0.501
	rh_OL—rh_TL	−0.05	0.506	0.05	0.517	0.14	0.068	0.19	0.010
	rh_PL—rh_TL	−0.01	0.920	0.04	0.618	0.10	0.220	−0.04	0.593
Somato-motor	lh_FL—lh_PL	0.08	0.288	−0.02	0.842	0.02	0.842	−0.04	0.553
	lh_FL—rh_FL	0.08	0.314	−0.01	0.885	0.02	0.814	0.17	0.028
	lh_FL—rh_PL	0.10	0.204	−0.03	0.713	0.10	0.201	0.07	0.403
	lh_PL—lh_TL	−0.11	0.142	−0.05	0.523	−0.03	0.714	0.03	0.659
	lh_PL—rh_FL	0.03	0.711	0.06	0.450	0.07	0.389	−0.06	0.421
	lh_PL—rh_PL	0.02	0.828	−0.03	0.735	0.13	0.108	0.07	0.354
	rh_FL—rh_PL	0.09	0.247	0.11	0.137	0.15	0.050	0.11	0.133
	rh_PL—rh_TL	−0.12	0.107	0.05	0.546	−0.02	0.751	−0.06	0.429
Ventral attention	lh_FL—lh_PL	0.05	0.509	−0.02	0.786	−0.02	0.752	−0.07	0.325
	lh_FL—rh_FL	−0.04	0.602	0.13	0.079	−0.05	0.488	0.17	0.022
	lh_PL—lh_TL	0.01	0.862	0.09	0.256	0.13	0.107	0.13	0.108
	lh_PL—rh_PL	0.08	0.281	0.02	0.777	0.12	0.115	0.02	0.839
	rh_FL—rh_PL	−0.07	0.387	−0.06	0.460	0.00	0.962	0.04	0.612
	rh_PL—rh_TL	0.03	0.687	0.03	0.663	0.10	0.194	0.10	0.186
Limbic	lh_FL—lh_TL	−0.01	0.889	0.08	0.284	0.08	0.294	−0.06	0.422
	lh_FL—rh_FL	−0.19	0.015	0.15	0.054	0.15	0.044	0.01	0.944
	rh_FL—rh_TL	−0.07	0.346	0.06	0.416	0.04	0.637	−0.11	0.172
Frontal-parietal	lh_FL—lh_PL	0.05	0.530	0.00	0.999	0.03	0.665	0.09	0.266
	lh_FL—rh_FL	0.05	0.543	0.08	0.286	0.19	0.012	−0.07	0.358
	lh_PL—rh_PL	−0.01	0.882	0.08	0.313	0.07	0.339	0.02	0.779
	rh_FL—rh_PL	−0.12	0.113	0.04	0.643	0.02	0.790	0.09	0.211
	rh_PL—rh_TL	0.03	0.705	−0.04	0.581	−0.06	0.430	0.18	0.016
Default mode	lh_FL—lh_PL	0.05	0.550	0.02	0.812	0.02	0.764	0.02	0.821
	lh_FL—lh_TL	−0.05	0.499	−0.03	0.676	0.09	0.277	0.04	0.640
	lh_FL—rh_FL	0.04	0.635	0.09	0.230	0.08	0.276	0.06	0.447
	lh_PL—lh_TL	−0.05	0.500	0.17	0.026	0.12	0.123	0.14	0.070
	lh_PL—rh_PL	−0.09	0.222	0.06	0.402	0.14	0.057	0.05	0.501
	rh_FL—rh_PL	−0.16	0.040	−0.04	0.566	0.04	0.605	0.15	0.047
	rh_FL—rh_TL	−0.22	0.005	0.18	0.020	0.15	0.056	0.12	0.117
	rh_PL—rh_TL	−0.06	0.445	0.02	0.745	−0.02	0.743	0.11	0.130

*lh, left hemisphere; rh, right hemisphere; FL, frontal lobe; OL, occipital lobe; PL, parietal lobe; TL, temporal lobe*.

The relationship between SC and FC has been explored previously within younger and elderly healthy participants in different studies (Andrews-Hanna et al., 2007; Fjell et al., 2015b; Marstaller et al., 2015; Hirsiger et al., 2016), however, results have been inconsistent. The first study by Andrews-Hanna et al. demonstrated a significant positive linear relationship between FC and FA (FC measured for the prefrontal cortex and retrosplenial/posterior cingulate cortex in the default mode network, and FA measured in a large WM region that included tracks connecting anterior to posterior regions) in elderly subjects. Similarly, subsequent studies by Fjell et al. and Marstaller et al. demonstrated that in younger subjects, FA of the uncinate was negatively correlated with FC between hippocampus and cortical regions (Fjell et al.), and global FA was negatively correlated with FC in the prefrontal regions of frontal-parietal and salience networks (FPN and SN; Marstaller et al.). In elderly subjects, MD of the cingulate bundle was positively correlated with FC between caudate and cortical regions (Fjell et al.), and global MD was positively correlated with FC in the prefrontal regions of FPN and SN (Marstaller et al.). While these studies demonstrated significant relationships between FC and FA or MD, a recent study by Hirsiger et al. did not find any significant relationships between FC (measured between posterior cingulate cortex and medial prefrontal cortex) and either AD, MD, RD, or FA (measured from the cingulum bundle) in healthy elderly subjects. During the preparation of this manuscript, another study similar to our present study was published (Fjell et al., 2016) and demonstrated modest relationship between SC and FC measures only within certain regions of the default mode network. It should be pointed out that the results reported by Fjell et al. were essentially derived from data obtained between two age groups of participants between 20 and 40 years and above 60 years, with only one participant around 50 years of age.

The results from the present cross-sectional study across the adult lifespan with relatively even number of participants in each decade of adulthood, rather than comparing between groups of elderly and younger subjects, show that the change in WM microstructure is not significantly related to the corresponding change in FC within the seven RSNs tested, contrary to our expectation.

### Study limitations

This study has a number of limitations. First there is a gender imbalance, which is most pronounced in the middle aged (40–49 years) and elderly (>60 years) groups. Sex differences were observed in SC in most networks, but there were no significant age-sex interactions. Therefore, we believe that results are generalizable to both women and men, though future studies should investigate sex differences further. Second, the question related to whether WM microstructural changes in normal aging precede FC changes cannot be addressed in this cross-sectional study. Future longitudinal studies will need to test age-related changes within individuals to better elucidate the relationships of SC and FC. Third, we used template-defined RSNs that do not allow for potential changes in the topography of these networks with age. It would be an interesting avenue for future study to incorporate spatial and temporal lifespan changes into networks defined by age group or at an individual level. Furthermore, we have not comprehensively investigated the relationship of SC and FC in all RSNs, for example, the salience network that was not part of the template was not included in the analysis. Future studies are needed to explore other networks that have been omitted in this work. Finally, despite the importance of DTI based tractography algorithms to provide quantitative measures to characterize WM microstructure integrity and architecture, these algorithms are limited to provide accurate delineation of the anatomical structural connections (Thomas et al., 2014). This latter observation may lead to spurious or missed WM tracts that belong to RSNs. Furthermore, one difference across studies of SC and FC relates to the choice of tractography algorithm used. It was shown recently that both deterministic and probabilistic tractography algorithms yielded similar relationships between SC with FC in regions associated with the default mode network (Khalsa et al., 2014). However, another study showed that both deterministic and probabilistic tractography algorithms underestimated the corticospinal tract connections to the sensorimotor cortex but a more complex algorithm based on constrained spherical deconvolution (CSD) reliably delineate the tracts that closely resembled to known anatomy of that brain region (Farquharson et al., 2013). Future studies should compare different tractography approaches and evaluate the impact on the relationships between SC and FC in normal aging.

## Conclusions

A multi-modal analysis approach using DTI and rs-fMRI data was used to investigate SC and FC within seven commonly described RSNs. SC measures demonstrated significant age-related changes in all networks, while FC demonstrated significant age-related changes in four of the seven networks. Despite significant age correlations in both SC and FC parameters, however, these were in general not significantly related to each other, suggesting that the change in WM microstructure measures with age is too weak to reflect the corresponding cortical FC change in resting-state networks. These results help further understand healthy brain aging, and lay the foundation for future studies to investigate age-related changes in connectivity in adults with neurodegenerative diseases.

## Ethics statement

This study was carried out in accordance with the recommendations of University of Calgary Conjoint Health Research Ethics Board (CHREB) with written informed consent from all subjects. All subjects gave written informed consent in accordance with the Declaration of Helsinki. The protocol was approved by the CHREB.

## Author contributions

AT performed analyses and wrote the manuscript. CL, SB, BG, and RS provided guidance in DTI tractography and resting state fMRI analyses. MH assisted in data processing. CM and RF designed study and reviewed manuscript.

### Conflict of interest statement

The authors declare that the research was conducted in the absence of any commercial or financial relationships that could be construed as a potential conflict of interest.
